# *Bacillus* sp. probiotic supplementation diminish the *Escherichia coli* F4ac infection in susceptible weaned pigs by influencing the intestinal immune response, intestinal microbiota and blood metabolomics

**DOI:** 10.1186/s40104-019-0380-3

**Published:** 2019-09-12

**Authors:** Diana Luise, Micol Bertocchi, Vincenzo Motta, Chiara Salvarani, Paolo Bosi, Andrea Luppi, Flaminia Fanelli, Maurizio Mazzoni, Ivonne Archetti, Giuseppe Maiorano, Bea K. K. Nielsen, Paolo Trevisi

**Affiliations:** 1 Department of Agricultural and Food Sciences, University of Bologna, Viale G. Fanin 46, 40127 Bologna, Italy; 2 Department of Agricultural, Environmental and Food Sciences, University of Molise, Via F. De Sanctis, Campobasso, Italy; 3 Istituto Zooprofilattico Sperimentale della Lombardia e dell’Emilia Romagna Bruno Ubertini, V. Bianchi 9, 25124 Brescia, Italy; 4 Endocrinology Unit and Center for Applied Biomedical Research, Department of Medical and Surgical Sciences, University of Bologna – S.Orsola-Malpighi Hospital, via Massarenti 9, 40138 Bologna, Italy; 5 Department of Veterinary Medical Sciences, University of Bologna, Via. Tolara di Sopra 50, 40064 Ozzano Emilia, Italy; 6 Chr. Hansen A/S, Boege Allé 10-12, 2970 Hoersholm, Denmark

**Keywords:** *Bacillus amyloliquefaciens*, *Bacillus subtilis*, Diarrhea, ETEC, Gut microbiota, Post-weaning, Transcriptomics

## Abstract

**Background:**

Probiosis is considered a potential strategy to reduce antibiotics use and prevent post-weaning diarrhea (PWD). This study investigated the effect of *Bacillus amyloliquefaciens* DSM25840 or *Bacillus subtilis* DSM25841 supplementation on growth, health, immunity, intestinal functionality and microbial profile of post-weaning pigs after enterotoxigenic *E. coli* (ETEC) F4 challenge.

**Methods:**

Sixty-four post-weaning piglets (7748 g ± 643 g) were randomly allocated to four groups: control basal diet (CO); CO + 1.28 × 10^6^ CFU/g of *B. amyloliquefaciens* (BAA); CO + 1.28 × 10^6^ CFU/g feed of *B. subtilis* (BAS); CO + 1 g colistin/kg of feed (AB). At day (d) 7, animals were challenged with 10^5^ CFU/mL of ETEC F4ac O149 and then followed for fecal score and performance until d 21. Blood was collected at d 6, d 12 and d 21 for immunoglobulins, at d 8 for acute phase proteins, at d 8 and d 21 for metabolomics analysis. Jejunum was sampled for morphometry, quantification of apoptosis, cell proliferation, neutral and acid mucine and IgA secretory cells, and microarray analysis at d 21. Jejunum and cecum contents were collected for microbiota at d 21.

**Results:**

AB and BAS reduced the fecal score impairment compared to CO (*P* < 0.05) at d 14. Body weight (BW), average daily weight gain (ADWG), average daily feed intake (ADFI) and gain to feed ratio (G:F) did not differ between *Bacillus* groups and CO. AB improved BW at d 7, d 14 and d 21, ADWG ADFI and G:F from d 0 to d 7 (*P* < 0.05). At d 8, CO had higher plasma arginine, lysine, ornithine, glycine, serine and threonine than other groups, and higher haptoglobin than AB (*P* < 0.05). At d 21, CO had lower blood glycine, glutamine and IgA than BAS. Morphology, cells apoptosis and mucins did not differ. BAS and AB increased the villus mitotic index. Transcriptome profile of BAS and AB were more similar than CO. Gene sets related to adaptive immune response were enriched in BAA, BAS and AB. CO had enriched gene set for nuclear structure and RNA processing. CO had a trend of higher Enterobacteriaceae in cecum than the other groups (*P* = 0.06).

**Conclusion:**

*Bacillus subtilis* DSM25841 treatment may reduce ETEC F4ac infection in weaned piglets, decreasing diarrhea and influencing mucosal transcriptomic profile.

**Electronic supplementary material:**

The online version of this article (10.1186/s40104-019-0380-3) contains supplementary material, which is available to authorized users.

## Introduction

Weaning remains a critical phase in pig production and it is related to the PWDS that cause digestive disorders and in some cases mortality. The enterotoxigenic *Escherichia coli* (ETEC) that express the F4 ac fimbria is considered one of the main etiological pathogens associated with post-weaning diarrhea syndrome (PWDS) in piglets [1].

Antibiotics have long been added in the starter diet of piglets, however, their continued use is an ongoing growing concern for the occurrence of antimicrobial resistance. Therefore, the study of alternatives to antibiotics is important for the feed industry, for pig farmers and stakeholders to develop a more sustainable production system less dependent on antibiotic use. Among the alternatives, probiotic bacteria have been investigated for their preventive role in contrasting the ETEC infection by the modulation of the gastrointestinal microbiota, favouring the gut eubiosis [2–4], or by their potential immunomodulatory effect on the intestinal immune response [5, 6]. Among probiotics, those of *Bacillus* group *in sensu lato* are considered promising beneficial bacteria for their capacity to produce antimicrobial substances such as bacteriocins, peptides and lipopeptides [7]. However, different strains from one species can have very different properties [8]. Recently, *Bacillus amyloliquefaciens* DSM25840 (*B. amyloliquefaciens)* and *Bacillus subtilis* DSM25841 (*B. subtilis*) have been ascribed as potential probiotic for pigs based on extensive *in vitro* screening [8] and have shown positive effects in production trials in piglets [9]. While no influence on nursery piglets’ performance supplemented whit a mixture of *B. licheniformis*, *B. subtilis* and *B. amyloliquefaeceans* spores has been observed by Poulsen et al. [10], other studies have shown that also other strains of *B. subtilis* and *B. amyloliquefaciens* exhibited promising activity against pathogenic bacteria [7]. In pigs, the administration of *B. amyloliquefaciens* increased body weight, reduced diarrhea incidence and enhanced the antioxidant status of weaned piglets [11, 12]. Furthermore, it showed an improvement of the mucosal morphology, a decrease of tumour necrosis factor alpha (TNFα) level and a beneficial regulation of the microbiota in the small intestine of intra-uterine growth retardation (IUGR) piglets [13]. *B. subtilis* supplementation has been tested as probiotic giving promising results in sows and suckling piglets [14], in growing pigs [15] and in growing pigs in a mix with *B. licheniformis* [16] and in weaned [17] and suckling [18] piglets.

Although previous studies demonstrated the probiotic role of *Bacillus* strains, only a few studies investigated the effect of *Bacillus* strains against diarrhea caused by ETEC in weaned piglets [19, 20]. Therefore, the aims of the present study were to evaluate the ability of dietary administration of *B. amyloliquefaciens* DSM25840 or *B. subtilis* DSM25841 in counteracting the ETEC F4ac infection of weaned piglets and to investigate the mode of action of these specific *Bacillus* strains on piglets’ health and intestinal functionality.

## Material and methods

### Animals and experimental desing

In total 64 piglets were selected from a farm where ETEC F4ac infection was frequently evidenced and based on the polymorphism for the *mucin4* gene [21], in order to obtain ETEC F4ac susceptible pigs [1]. At weaning [24 ± 2 days of age; initial body weight (BW) 7.75 kg ± 0.64 kg] (d 0) animals were moved to the experimental facility of the Department of Agricultural and Food Science (DISTAL) - University of Bologna. Pigs were housed in individual cages with a mesh floor except for the first 3 days of the trial when pigs were kept in groups of two animals to stimulate the feed intake immediately after weaning. Room temperature was kept controlled at 30 °C at the beginning and 25 °C at the end of the experiment, with a 1 °C decrease every 3 days. Infrared lamps were located above the piglets for the first 7 days post-weaning. The piglets had free access to feed and water throughout the experimental period; feed was ad libitum supplied in a dry feeder. At d 0 pigs were assigned to four different groups (16 piglets/group) balanced by litter and BW. The groups were assigned to 1 of the 4 treatments: i) Control group (CO) fed with a basal diet (Table 1); ii) (BAA): CO + 1.28 × 10^6^ CFU/g feed *of B. amyloliquefaciens* DSM25840; iii) (BAS): CO + 1.28 × 10^6^ CFU/g feed of *B. subtilis* DSM25841; iv) antibiotic group (AB): CO + 1 g colistin/kg of feed.

**Table 1:** Ingredient and calculated composition of the basal diet (g/kg as fed basis)

Items	Content
Ingredients, %	
Corn	27.00
Barley	25.10
Biscuit by-product	20.00
Rice protein concentrate	4.20
Milk whey	4.00
Wheat middlings	4.00
Soybean meal, 50% CP	3.00
Wheat gluten	2.50
Soy oil	3.50
Brewer’s yeast	2.00
Potato, protein concentrate	1.50
Dicalcium phosphate	1.00
Carrob pulp	1.00
Miner-vitamin premix	0.50
Calcium carbonate	0.50
Salt	0.20
Calculated chemical composition	
Crude protein, %	16.50
Crude fat, %	5.80
Crude fibre, %	3.12
Ash, %	4.22
Ca, %	0.60
P, %	0.50
NaCl, %	0.38
Cu, mg	150
Zn, mg	135
Lysine,%	1.20
Met + Cys, %	0.70
ME, kcal/kg	3144

*ME* Metabolic energy, the values were estimated by the EvaPig® software [22] using information from the INRA-AFZ tables of feedstuff composition (INRA-AFZ, 2004)

On d 7, all pigs were orally dosed with 1.5 mL suspension containing 10^5^ CFU of ETEC F4ac O149 per mL. On d 8, 24 h after the ETEC infection, 24 animals (6 animals per group, randomly chosen), were deeply anaesthetized with sodium thiopental (10 mg/kg BW) and sacrificed by an intracardiac injection of Tanax® (0.5 mL/kg BW). The remained piglets were kept and followed until d 21 and then sacrificed using the same procedure.

### Sampling and data collection

Body weight was recorded weekly from the beginning of the trial (d 0) to the slaughtering (d 21). In the first 2 days, feed intake was measured for the groups of two pigs. From d 3, when pigs started to be individually penned, individual feed intake of each pig was registered every day.

For each pig, the severity of diarrhea was recorded daily from d 4 to d 21 using a 5-point fecal score system: 1 = hard; 2 = firm; 3 = soft (moist stool); 4 = soft (unformed stool); 5 = watery faeces [1, 23]. Pigs were excluded from the trial due to a severe impairment of health status when a fecal score of 5 was recorded for more than 4 continuous days and pigs showed low feed intake for the same period.

Plasma samples were individually collected at d 8 and d 21 using a K3 EDTA (Vacutest Kima Srl, Arzergrande PD Italy) collection tube by venipuncture of vena cava. To obtain plasma, blood samples were centrifuged at 3,000×*g* for 10 min at 4 °C. Plasma samples were stored at − 80 °C until further targeted metabolomics profile analysis [24].

Serum samples were individually collected by venipuncture of vena cava at d 6 (before challenge), d 12 and d 21, using a collection tube without anticoagulant. To obtain serum, blood was incubated at room temperature for 2 h, then centrifuged at 3,000×*g* for 10 min. The serum was incubated at 56 °C for 30 min and finally stored at − 20 °C for immunoglobulins and acute phase proteins analysis.

At d 21, after animals’ sacrifice, the gastrointestinal tract (GIT) was immediately removed. The mucosa from the distal part of jejunum (75% of the small intestine length) was gently scraped and snap-frozen in liquid nitrogen and then preserved at − 80 °C for microarray analysis. For microbiota analysis, the intestinal content from the same jejunum site and from cecum was immediately collected into a sterile tube, snap-frozen in liquid nitrogen and then preserved at − 80 °C. Furthermore, an additional intestinal segment from the same jejunum section was collected for morphometric, histology and immunohistochemistry analysis. For the morphometric analysis, tissue samples were fixed overnight in 10% neutral buffered formalin and embedded in paraffin. The sections were stained with hematoxylin-eosin for the morphometric evaluation.

### Plasma targeted metabolomics analysis

Plasma metabolites were measured using the Biocrates AbsoluteIDQTM p180 Kit (Biocrates Life Science AG, Innsbruck, Austria) that allows to quantify a panel of 188 compounds, including 40 acylcarnitines, 21 amino acids, 21 biogenic amines, 90 glycerophospholipids [14 lysophosphatidylcholines (lysoPC) and 76 phosphatidylcholines (PC)], 15 sphingolipids and hexoses (sum of hexoses, including glucose). The kit was processed according to manufacturer instructions and analysed on the Serie 200 high-pressure liquid chromatography system by Perkin Elmer (Waltham, MA) coupled with the API 4000 QTRAP by AB Sciex (Foster City, CA, USA). Amino acid and biogenic amine classes were analysed by liquid chromatography-tandem mass spectrometry (LC-MS/MS). Acylcarnitines, phospho- and sphingolipids and hexose were analysed by flow injection analysis (FIA) – MS/MS. Instrumental data were acquired and processed by Analyst 1.6.3, whereas data quantitation and validation were performed by MetIDQ-5.5.4-dB100-Boron-2623 software. Results were exported in micromolar unit (μmol/L).

### Serum immunoglobulins and acute phase protein determination

Serum IgA, IgG and IgM were quantified on samples collected at d 6, d 12 and d 21. Samples were diluted 1:6,400 for IgA, 1:500,000 for IgG and 1:50,000 for IgM. Determination of total IgA in serum was carried out by ELISA, using Pig Immunoglobulin Reference Serum (Bethyl Laboratories, Montgomery, TX) as the specific antibody for the standard curve, Goat anti-Pig IgA-HRP coniugate (Bethyl Laboratories) as a secondary antibody, and 2,2′-azino-bis (3-ethylbenzthiazoline-6-sulfonic acid; Roche Diagnostics, San Francisco, CA) for chromogenic detection as described by Bosi et al. [25]. Porcine specific commercial ELISA kits were used to analyse serum concentration of total IgM, IgG (Bethyl Laboratories, Montgomery, TX) according to the manufacturer’s instructions. Serum acute protein determination was carried out on the samples on d 8. Haptoglobin (Hp), Serum Amyloid A (SAA) and C-Reactive Protein (CRP) were determined using porcine specific commercial ELISA kits (Tridelta Development Limited, Maynooth, County Kildare, Ireland) according to the manufacturer’s instructions. Samples were diluted 1:3 for Hp, 1:50 for SAA and 1:100 for CRP. IgM, IgG and Hp concentrations were expressed as milligram per millilitre (mg/mL); SAA and CRP were expressed as milligram per litre (mg/L).

### Microbiota analysis

Total bacterial DNA was extracted using the FastDNA SPIN Kit for Soil (MP Biomedicals, Santa Ana, CA, USA) following the manufacturer’s instructions. The library formation and sequencing of the 16S rRNA gene were performed with MiSeq® Reagent Kit V3-V4 on MiSeq-Illumina® platform. Generated sequences were analysed using the subsampled open reference operational taxonomic unit (OTU) strategy with default settings in QIIME (v1.9.1). The paired-end reads were merged and demultiplexed. Subsampled open-reference OTU-picking was performed using UCLUST with 97% sequence similarity. Representative sequences were chimera checked using Blast_fragments approach with default settings and taxonomy assigned against the Greengenes database V13_8 using the UCLUST method with a 90% confidence threshold. The singletons and OTUs with relative abundance across all samples below 0.005% were excluded as recommended by Bokulich et al. [26]. The samples that reported low sequencing yield (less than 5,000 reads after quality check) were excluded from the analysis.

### RNA isolation and microarray analysis

For microarray analysis, 6 out 10 pigs per group were randomly selected. Total mRNA was isolated from 50 mg of frozen distal jejunum mucosa according to the Takara Fast Pure kit (Takara Bio, Japan) protocol. The purity and concentration of the total RNA were checked using the Nanodrop ND 1000 (Nanodrop Technologies, USA), while RNA integrity was assessed by Agilent Bioanalyzer 2100 (Agilent Technologies, USA). Total mRNA was hybridized on Affymetrix Porcine Gene 1.1 ST array strips. Hybridized arrays were scanned on a GeneAtlas imaging station (Affymetrix, Santa Clara, CA, USA). Performance quality tests of the arrays including the labelling, hybridization, scanning and background signals by a Robust Multichip Analysis were performed on the CEL files using the Affymetrix Expression Console. The Affymetrix Transcripts ID’s, in general, characterized each one by several exonic sequences, were associated with 13,494 human gene names, based on *Sus scrofa* Ensembl (release 83, www.ensembl.org).

### Morphometric, histochemical and immunohistochemistry analysis

Paraffin sections were deparaffinised in xylene and stained with haematoxylin-eosin. The height and width of 10 villi and the depth and width of 10 crypts (randomly selected) were measured for each sample. The mucosal-to-serosal amplification ratio (M) was calculated as following: M = (villous surface + unit bottom − villous bottom)/unit bottom, where villous surface = π × (villous length × villous width), unit bottom = π × (villous width/2 + crypt width/2)^2^ and villous bottom = π × (villous width/2)^2^, according by Kisielinski et al. [27]. Well-orientated intestinal tissue sections were used to count the goblet cells per intestine villi or crypt of each piglet.

The combination of the alcian blue (ALB) pH 2.5 and the periodic acid-Schiff method (PAS) techniques were used as a means of distinguishing acid mucins from neutral mucins respectively [28]. Briefly, the ALB at a pH of 2.5 will stain all acid mucins deep blue, while the PAS technique stain the neutral mucins bright magenta. The cells that contain both neutral and acidic mucins may demonstrate a purple coloration. Mean number of PAS, ALB and combined PAS/ALB-stained goblet cells per villus and crypts were evaluated by measuring 10 villi and 10 crypts.

Paraffin sections (7 μm thick), mounted on poly-*L*-lysine-coated slides, were processed for immunohistochemistry to evaluating the cellular proliferation (cell proliferation antigen Ki67) and the IgA secreting cells. In addition, to determine the apoptotic cells, the Apoptosis Detection Kit (ApopTag® Plus peroxidase *in situ* kit, S701, Millipore, Temecula, CA, USA) was used.

Briefly, paraffin sections were deparaffinized and rehydrated; to unmask the antigenic sites, the slides were heated in sodium citrate buffer (pH 6.0) in a microwave. Endogenous peroxidase was blocked with 1% aqueous hydrogen peroxide solution for 30 min at RT and subsequently incubated in PBS containing 10% normal goat serum to prevent nonspecific binding of the antibodies. The sections were then incubated overnight at 4 °C with the following antibodies: mouse anti-Ki67 antibody diluted 1:700 (AB15580, Abcam, UK) and goat anti-porcine IgA 1:4000 (NB724, Novus Biological Abingdon, UK). After washing, sections were incubated for 1 h with the appropriate biotin-conjugated secondary antibody [goat anti-mouse IgG and horse anti-goat IgG, both diluted 1:500 (Vector)] and then treated with ABC complex (Vector elite kit, Vector Laboratories). The immune reactions were visualized applying a 3,3′-diaminobenzidine chromogen solution (Vector DAB kit, Vector Laboratories). IgA quantitation was calculated according to the method of Waly et al. [29]: five appropriate areas were randomly chosen for each piglet; the areas were delineated on the computer screen, excluding the epithelium, large blood vessel and artefacts; positively stained cells within each region were counted. Results were expressed as positive cells per 10,000 μm^2^.

### Statistical analysis and data processing

For mortality data, Fisher’s exact test was performed testing separately BAA, BAS and AB against CO. Data of performances, fecal score, Igs, acute phase proteins, plasma metabolites and morphometric and immune histological data were analysed by analysis of variance using the GLM procedure of SAS (SAS Inst., inc., Cary, NC) considering the treatment and the origin litter as factors. Sex was initially included as a fixed factor but resulted always not significant and was dropped off the final model. The available degrees of freedom for the treatment were used for testing separately BAA, BAS and AB against CO. Before statistical analysis, metabolomics data were imported into the MetaboAnalyst 3.0 software [30] where data integrity was checked. Metabolites showing more than 50% of missing values were removed. Data normalization were performed against the median of metabolite concentrations measured for the treatment group, then data were log transformed and mean-central scaled. *P*-values were corrected for false discovery rate (FDR). *P*-value < 0.05 was considered as significant and *P*-value < 0.1 was considered a trend of significance.

Biostatistics on OTUs table were performed using phyloSeq [31], Vegan [32] packages in R software (v.3.3.0). The richness and alpha diversity indices (Shannon and Chao indices) were calculated on the raw data matrix while beta diversity ordination (Bray-Curtis distance matrix) were calculated after rarefaction correction.

Alpha diversity indices were compared with multivariate ANOVA testing litters and the treatment as explanatory variables. The treatment differences in beta diversity were explored using permutational manova (Adonis procedure) on the Bray-Curtis distance matrix. Then, in order to evaluate differences in the most abundant family among groups the Wilcoxon signed-rank test was performed. *P*-values were corrected for FDR correction. *P*-value < 0.05 was considered as significant and *P*-value < 0.1 was considered a trend of significance.

On processed gene expression values, an exploratory functional analysis was performed using the Gene Set Enrichment Analysis (GSEA) approach. The GSEA approach is based on gene sets, defined as groups of genes with common biological function, chromosomal location, or regulation [33]. The GSEA analysis was carried out on Gene Set Enrichment Analysis software, using the C5.v5.1 catalogue of gene sets (based on Gene Ontology) from Molecular Signatures Database v3.1 (http://software.broadinstitute.org/gsea/msigdb/index.jsp). The normalized enrichment score (NES) was calculated for each gene set. BAA, BAS and AB treatments were separately tested against CO and statistical significance was considered when false discovery rate (FDR) % < 25 and *P*-values of NES < 0.05 [34]. Enrichment score *P*-values were estimated using a gene set-based permutation test procedure following the GSEA recommended pipeline.

The common up and down-regulated gene sets among AB, BAA and BAS in contrast with the CO group were visualized using a Venn diagram performed using Venny software (http:// bioinfogp.cnb.csic.es/tools/venny/).

## Results

One subject of the AB group was excluded from the trial because injured during the transport.

### Monitoring of health status

No significant differences in the number of piglets excluded from the trial for severe impairment of health status were observed between *Bacillus* dietary supplementation groups and CO. The number of excluded piglets after the challenge was, however, higher in the CO group. In the AB group, all pigs remained healthy during the entire experimental period. In details: 2 piglets from BAA group, 1 piglet from BAS group, and 1 piglet from CO group were excluded during the first week, before ETEC F4 challenge. Twenty-four hours after the infection (d 8), 2 piglets of the BAA group and 1 piglet of the CO group were excluded. During the period from d 10 to d 12, 3 additional pigs of CO group were excluded. The CO group had a higher fecal score than the AB group (*P* ≤ 0.01), while BAS tended to reduce the fecal score at d 14 compared to CO (*P* = 0.06). No differences in fecal score were observed between CO and BAA groups (Table 2).

**Table 2:** Effect of dietary supplementation of *Bacillus amyloliquefaciens* DSM25840 and *Bacillus subtilis* DSM25841 on fecal score and days of diarrhea of weaned pigs challenged with *Escherichia coli* F4ac

Items	Treatments^a^				SEM	*P*-value		
	BAA	BAS	AB	CO		BAA vs. CO	BAS vs. CO	AB vs. CO
Fecal score								
Day 6^b^	3.75	3.72	1.97	3.63	0.27	0.45	0.54	< 0.001
Day 8^c^	3.66	3.44	1.9	3.38	0.28	0.28	0.68	< 0.001
Day 14^d^	3	2.4	1.78	3.4	0.38	0.4	0.06	0.01
Day 21^d^	3.15	2.6	1.89	3.4	0.34	0.56	0.11	0.01

^a^BAA *B. amyloliquefaciens* DSM25840, BAS *B. subtilis* DSM25841, AB Antibiotic, CO Control

^b^Number of animals was 16 for BAA, BAS and CO groups; 15 for AB group

^c^Number of animals was: 14 for BAA; 15 for BAS, AB and CO.

^d^Number of animals was 6 for BAA; 9 for BAS; 10 for AB; 6 for CO

### Performances

No differences for BW and ADWG at d 7, d 14 and d 21 were observed for the BAA and the BAS groups compared to the CO group (Table 3). The AB group had higher BW at d 7, d 14 and d 21 than the CO group did (*P* ≤ 0.05). Moreover, the AB group had higher ADWG than the CO group during the first-week post-weaning (d 0 to d 7) (*P* < 0.001), and a trend of higher ADWG was observed in the post-challenge period (d 8 - d 21) in the AB group than in the CO group (*P* = 0.09).

**Table 3:** Effect of dietary supplementation of *Bacillus amyloliquefaciens* DSM25840 and *Bacillus subtilis* DSM25841 on growth performance of weaned pigs challenged with *Escherichia coli* F4ac

Items	Treatments^a^				SEM	*P*-value		
	BAA	BAS	AB	CO		BAA vs. CO	BAS vs. CO	AB vs. CO
Body weight, g								
Initial^b^	7779	7789	7869	7619	266	0.60	0.60	0.49
Day 7^c^	7638	7581	8208	7356	308	0.64	0.60	0.02
Day 14^d^	8912	8279	9201	8370	350	0.33	0.92	0.05
Final (day 21)	10855	10036	11319	10102	549	0.28	0.94	0.03
Average daily live weight gain, g/d								
Day 0 - day 7^c^	−27.6	−41.6	48.5	−62.3	28.7	0.61	0.99	< 0.001
Day 7 - day 14^d^	130	108	138	111	29.8	0.32	0.65	0.30
Day 14 - day 21^d^	278	251	303	248	35.4	0.46	0.75	0.12
Post-challenge (day 8 - day 21)^d^	204	179	220	179	29.1	0.27	0.63	0.09
Total (day 0 - day 21)^d^	129	99	156	109	28.3	0.51	0.60	0.10
Average daily feed intake, g/d								
Day 0 - day 7^c^	162	163	200	146	15.6	0.70	0.34	< 0.001
Day 7 - day 14^d^	308	254	376	318	46.8	0.78	0.27	0.32
Day 14 - day 21^d^	535	532	612	519	47.1	0.75	0.71	0.07
Post-challenge (day 8 - day 21) ^d^	421	393	494	418	44.6	0.99	0.65	0.11
Total (day 0 - day 21) ^d^	329	318	396	330	33.3	0.82	0.72	0.06
Gain to feed								
Day 0 - day 7^c^	−0.52	−0.38	0.19	−0.56	0.19	0.62	0.76	< 0.001
Day 7 - day 14 ^d^	0.38	0.37	0.23	0.34	0.11	0.54	0.61	0.57
Day 14 - day 21 ^d^	0.51	0.46	0.49	0.47	0.03	0.35	0.93	0.39
Post-challenge (day 8 - day 21) ^d^	0.46	0.44	0.44	0.42	0.03	0.06	0.11	0.22
Total (day 0 - day 21) ^d^	0.36	0.28	0.38	0.30	0.05	0.22	0.74	0.20

^a^BAA *B. amyloliquefaciens* DSM25840, BAS *B. subtilis* DSM25841, AB Antibiotic, CO Control

^b^Number of animals was 16 for BAA, BAS and CO groups; 15 for AB group

^c^Number of animals was: 14 for BAA; 15 for BAS, AB and CO.

^d^Number of animals was: 6 for BAA; 9 for BAS; 10 for AB; 6 for CO

No differences for ADFI were observed for BAA and BAS groups compared to the CO group. The AB group had higher ADFI than the CO group during first-week post-weaning (d 0 to d 7) (*P* < 0.001). A trend of higher ADFI was observed in the AB group than in the CO group for the period d 8 - d 14 and for the global period. No differences for G:F were observed for the BAS group compared to the CO group. The AB group had higher G:F than CO group (*P* < 0.001) during first-week post-weaning (d 0 to d 7) and a trend of higher G:F was observed in the BAA group than in the CO group for post-challenge (d 8 - d 21) period (*P* = 0.06).

### Plasma targeted metabolomics

The MS/MS targeted analysis provided the quantification of 138 metabolites (Additional file 1: Table S1 SS1). Significantly different metabolites in the blood plasma at d 8 and d 21 are reported in Table 4. At d 8 the BAA group showed lower concentration of arginine [adjiusted *P* (*P*_adj_) = 0.02], lysine (*P*_adj_ = 0.03) and ornitine (*P*_adj_ = 0.04) and a trend of lower concentration of threonine (*P*_adj_ = 0.06), histamine (*P*_adj_ = 0.07) and glycine (*P*_adj_ = 0.08) than CO group. The BAS group showed lower concentration of lysine (*P*_adj_ = 0.03), glycine (*P*_adj_ = 0.02), serine (*P*_adj_ = 0.05) and P.aa.C30.0 (*P*_adj_ = 0.05), and a trend for lower concentration of proline (*P*_adj_ = 0.07) than CO group did on plasma at d 8. The AB group had a lower concentration of arginine (*P*_adj_ = 0.02), lysine (*P*_adj_ = 0.02) and threonine (*P*_adj_ = 0.05) than CO group did on plasma at d 8.

**Table 4:** Effect of dietary supplementation of *Bacillus amyloliquefaciens* DSM25840 and *Bacillus subtilis* DSM25841 on the blood plasma metabolites showing statistical significances in weaned piglets challenged with *Escherichia coli* F4ac

Metabolite, μmol/L	Treatments^a^				SEM	*P*-value		
	BAA	BAS	AB	CO		BAA vs. CO	BAS vs. CO	AB vs. CO
Day 8^b^								
Arg	107.0	117.1	105.2	145.3	16.8	0.02	0.11	0.02
Gly	657.3	597	785.7	893.7	72.3	0.08	0.02	0.64
Lys	191.5	225.7	188.3	246.3	19.3	0.03	0.03	0.02
Orn	58.0	65.4	63.8	82.9	11.0	0.04	0.19	0.14
Pro	228.5	216.5	242.5	292.6	23.6	0.14	0.07	0.31
Ser	111.1	101.7	116.4	133.1	6.8	0.22	0.05	0.44
Thr	91.1	123.5	78.1	180.4	28.0	0.1	0.41	0.05
Tyr	51.2	57.8	53.1	69.4	5.0	0.06	0.34	0.11
Histamine	153.4	73.1	81.5	73.4	12.5	0.07	0.85	0.98
PC.aa.C30.0	1.2	1.1	1.4	1.4	0.1	0.27	0.05	0.93
Day 21^c^								
Gly	1058	927.8	910.1	791.2	55.8	0.04	0.39	0.5
Gln	551	482.6	502.3	454.7	25.8	0.05	0.82	0.46
Ser	154.7	138.9	143.7	113.6	9.9	0.07	0.31	0.18
SDMA	0.7	1.0	0.9	1.3	0.2	0.07	0.51	0.22

^a^BAA *B. amyloliquefaciens* DSM25840, BAS *B. subtilis* DSM25841, AB Antibiotic, CO Control

^b^ Number of included animals was: 14 for BAA; 15 for BAS, AB and CO.

^c^ Number of included animals was: 6 for BAA; 9 for BAS; 10 for AB; 6 for CO

At d 21, the BAA group had a higher concentration of glycine (*P*_adj_ = 0.04) and glutamine (*P*_adj_ = 0.05) and a trend for a higher concentration of serine and a lower concentration of SDMA (*P*_adj_ = 0.07) than CO group did.

### Serum immunoglobulins and acute phase proteins

Table 5 reports the results for serum IgA, IgG and IgM at d 6, d 12 and d 21. At d 6, the BAA group had a higher IgM concentration (*P* = 0.04) and a trend of higher IgG concentration (*P* < 0.01) compared to CO. At d 12, no differences in serum Igs were observed among groups. At d 21, the BAS group had a higher IgA concentration than CO group did (*P* = 0.04). For the acute phase proteins, no differences in SAA and CRP levels were reported among groups at d 8. For Hp, the CO group had a higher serum Hp level than the AB group (*P* = 0.05; 4.50 mg/mL in CO and 3.20 mg/mL in AB).

**Table 5:** Effect of dietary supplementation of *Bacillus amyloliquefaciens* DSM25840 and *Bacillus subtilis* DSM25841 on the blood serum IgA, IgG and IgM of weaned piglets challenged with *Escherichia coli* F4ac

Igs	Treatments^a^				SEM	*P*-value		
	BAA	BAS	AB	CO		BAA vs. CO	BAS vs. CO	AB vs. CO
Day 6, mg/mL^b^								
IgA	0.41	0.46	0.37	0.49	0.10	0.84	0.98	0.63
IgG	19.80	13.72	13.92	13.54	2.61	0.09	0.96	0.91
IgM	3.88	2.85	2.84	3.06	0.28	0.04	0.59	0.55
Day 12, mg/mL^c^								
IgA	0.72	0.46	0.42	0.54	0.09	0.51	0.85	
IgG	17.94	13.6	12.57	15.84	2.24	0.55	0.48	0.31
IgM	3.75	3.96	3.03	3.38	0.54	0.66	0.45	0.64
Day 21, mg/mL^d^								
IgA	0.72	0.96	0.65	0.68	0.08	0.92	0.04	0.98
IgG	10.57	11.58	11.88	12.14	1.35	0.45	0.71	0.89
IgM	2.86	3.25	2.75	2.82	0.32	0.93	0.34	0.88

^a^BAA *B. amyloliquefaciens* DSM25840, BAS *B. subtilis* DSM25841, AB Antibiotic, CO Control

^b^Number of animals was 16 for BAA, BAS and CO groups; 15 for AB group

^c^Number of animals was: 14 for BAA; 15 for BAS, AB and CO.

^d^Number of animals was: 6 for BAA; 9 for BAS; 10 for AB; 6 for CO

### Microbiota profile

For the jejunum, a total of 502,700 quality checked reads were obtained, which returned a total of 475 different OTUs, distributed among the samples as shown in Additional file 2: Table S2 SS2. For the cecum, a total of 778,626 quality-checked reads were obtained, which returned a total of 1,179 different OTUs, distributed among the samples (Table SS2).

No significant effects of treatment were observed for the Shannon and Chao indices both in jejunum and in cecum. For Beta diversity (Bray-Curtis distance), the Adonis test showed no differences for BAA, BAS and AB compared to CO. The Non-Metric Multidimensional Scaling (NMDS) plot, performed on Bray-Curtis distance matrix, showed no defined cluster due to treatments (Additional file 3: Figure S1).

The jejunum was dominated by Lactobacillaceae (63% of relative abundance) followed by Streptococcaceae (14%) and Clostridiaceae (9%) families. Dominant families in cecum were Lactobacillaceae (23%), Prevotellaceae (21%) and Ruminococcaceae (19%), followed by Veillonellaceae (10%) and Lachnospiraceae (9%). Table 6 reports the results of Kruskal-Wallis test on microbial families of jejunum and cecum (only the Family with *P*-value < 0.05). No significant difference was observed in the jejunum. In cecum, a trend of higher abundance for the Enterobacteriaceae family was identified in CO compared to BAA, BAS and AB (*P*_adj_ = 0.06).

**Table 6:** Effect of the dietary supplementations of *Bacillus amyloliquefaciens* DSM25840 and *Bacillus subtilis* DSM25841 on abundance of microbial families of weaned piglet 14 days after the *Escherichia coli* F4ac challenge

Family	Treatments^a^				SEM	*P*-value	FDR, *P*- value
	BAA	BAS	AB	CO			
Jejunum							
Pasteurellaceae	1.53	1.07	0.01	13.62	0.12	0.024	1.189
Succinivibrionaceae	0.00	0.00	0.00	0.01	0.00	0.115	2.827
Moraxellaceae	0.00	0.00	0.00	0.02	0.00	0.135	2.199
Cecum							
Enterobacteriaceae	0.14	0.38	0.01	1.05	0.03	0.001	0.058
Ruminococcaceae	8.20	21.82	20.96	24.43	0.09	0.011	0.273
Peptostreptococcaceae	0.02	0.05	0.10	0.25	0.01	0.013	0.213

^a^BAA *B. amyloliquefaciens* DSM25840, BAS *B. subtilis* DSM25841, AB Antibiotic, CO Control. Data are reported as mean of relative abundance %

At genus level, the jejunum was dominated by *Lactobacillus* (61%) followed by *Streptococcus* (12%), *Sarcina* (19%) and *Actinobacillus* (1.50%) genera. Dominant genera in cecum were *Lactobacillus* (23%), *Prevotella* (21%), unclassified_*Ruminococcaceae* (12%) followed by *Faecalibacterium* (4.7%) and unclassified_*Lachnospiraceae* (4.5%). No significant different genera were observed between groups in jejunum and cecum.

### Transcriptomic profile

To discriminate specific effects of probiotic treatments, the enrichment gene set analysis based on Hallmark genes was performed. Tables 7, 8 and 9 show the most significantly enriched gene sets (FDR *q*-values ≤0.05) obtained by the comparison of CO group to BAA, BAS and AB groups, respectively.

**Table 7:** Effect of the dietary supplementations of *Bacillus amyloliquefaciens* DSM25840 on the transcriptomic profile of jejunum mucosa of weaned piglet 14 days after the *Escherichia coli* F4ac challenge

Name of the enriched gene set	Size, No. of genes	NES	*P*-value	FDR, *q*
Gene sets enriched in BAA respect to CO^a^				
STRUCTURAL_CONSTITUENT_OF_RIBOSOME	56	2.69	< 0.001	< 0.001
MITOCHONDRIAL_MEMBRANE_PART	40	2.22	< 0.001	< 0.001
MITOCHONDRIAL_RESPIRATORY_CHAIN	19	2.11	< 0.001	0.01
RIBOSOME	25	2.08	< 0.001	0.01
MITOCHONDRIAL_INNER_MEMBRANE	51	2.02	< 0.001	0.02
Gene sets enriched in CO respect to BAA^a^				
EXONUCLEASE_ACTIVITY	15	−2.20	< 0.001	< 0.001
EXTRACELLULAR_MATRIX_PART	42	−2.20	< 0.001	< 0.001
HELICASE_ACTIVITY	43	−2.20	< 0.001	< 0.001
PROTEINACEOUS_EXTRACELLULAR_MATRIX	74	−2.10	< 0.001	< 0.001
EXTRACELLULAR_MATRIX	75	−2.10	< 0.001	< 0.001
DNA_HELICASE_ACTIVITY	23	− 2.10	< 0.001	< 0.001
MEIOTIC_CELL_CYCLE	29	− 2.00	< 0.001	< 0.001
LIGASE_ACTIVITY	82	−2.00	< 0.001	< 0.001
CELL_CYCLE_PHASE	136	−2.00	< 0.001	0.01
DNA_REPAIR	105	−2.00	< 0.001	0.01
TRANSMEMBRANE_RECEPTOR_PROTEIN_TYROSINE _KINASE _ACTIVITY	39	−2.00	< 0.001	0.01
CHROMATIN_BINDING	26	−2.00	< 0.001	0.01
CELL_CYCLE_PROCESS	154	−2.00	< 0.001	0.01
DNA_DEPENDENT_DNA_REPLICATION	43	−2.00	< 0.001	0.01
M_PHASE	95	−2.00	< 0.001	< 0.001
COLLAGEN	17	−2.00	0.002	< 0.001
BASEMENT_MEMBRANE	28	−2.00	< 0.001	0.01
TRANSMEMBRANE_RECEPTOR_PROTEIN _KINASE_ACTIVITY	47	−2.00	< 0.001	0.01
RESPONSE_TO_DNA_DAMAGE_STIMULUS	135	−1.90	< 0.001	0.01
DNA_REPLICATION	69	−1.90	< 0.001	0.01

^a^BAA *B. amyloliquefaciens* DSM25840, CO Control

**Table 8:** Effect of the dietary supplementations of *Bacillus subtilis* DSM25841 on the transcriptomic profile of jejunum mucosa of weaned piglet 14 days after the *Escherichia coli* F4ac challenge

Name of the enriched gene set	Size, No. of genes	NES	*P*-value	FDR, *q*
Gene sets enriched in BAS respect to CO^a^				
RHODOPSIN_LIKE_RECEPTOR_ACTIVITY	97	2.66	< 0.001	< 0.001
MONOVALENT_INORGANIC_CATION_TRANSPORT	72	2.49	< 0.001	< 0.001
SODIUM_ION_TRANSPORT	17	2.43	< 0.001	< 0.001
CELL_CELL_SIGNALING	309	2.4	< 0.001	< 0.001
CATION_CHANNEL_ACTIVITY	90	2.37	< 0.001	< 0.001
SUBSTRATE_SPECIFIC_CHANNEL_ACTIVITY	119	2.3	< 0.001	< 0.001
ION_CHANNEL_ACTIVITY	112	2.27	< 0.001	< 0.001
G_PROTEIN_COUPLED_RECEPTOR_ACTIVITY	140	2.25	< 0.001	< 0.001
HORMONE_ACTIVITY	35	2.2	< 0.001	0.01
RESPONSE_TO_BACTERIUM	22	2.19	< 0.001	0.01
CYCLIC_NUCLEOTIDE_MEDIATED_SIGNALING	81	2.17	< 0.001	0.01
PEPTIDE_RECEPTOR_ACTIVITY	36	2.16	< 0.001	0.01
G_PROTEIN_SIGNALING_COUPLED_TO_CYCLIC_NUCLEOTIDE _SECOND_MESSENGER	79	2.14	< 0.001	0.01
VOLTAGE_GATED_CATION_CHANNEL_ACTIVITY	52	2.13	< 0.001	0.01
INORGANIC_ANION_TRANSPORT	16	2.13	0.005	0.01
GATED_CHANNEL_ACTIVITY	95	2.12	< 0.001	0.01
POTASSIUM_ION_TRANSPORT	45	2.11	< 0.001	0.01
VOLTAGE_GATED_POTASSIUM_CHANNEL_COMPLEX	32	2.08	< 0.001	0.01
G_PROTEIN_SIGNALING_COUPLED_TO_CAMP_NUCLEOTIDE_SECOND_MESSENGER	51	2.08	< 0.001	0.01
CATION_TRANSPORT	119	2.07	< 0.001	0.01
Gene sets enriched in CO respect to BAS^a^				
MICROTUBULE_ORGANIZING_CENTER	47	−2.2	< 0.001	< 0.001
CENTROSOME	39	−2.2	< 0.001	< 0.001
MRNA_METABOLIC_PROCESS	55	−2.1	< 0.001	< 0.001
LIGASE_ACTIVITY_FORMING_CARBON_NITROGEN_BONDS	56	−2.1	< 0.001	< 0.001
NUCLEOPLASM	227	−2.1	< 0.001	< 0.001
ACID_AMINO_ACID_LIGASE_ACTIVITY	46	−2.1	< 0.001	< 0.001
MICROTUBULE_CYTOSKELETON	110	− 2.1	< 0.001	< 0.001
LIGASE_ACTIVITY	82	−2	< 0.001	< 0.001
NUCLEOPLASM_PART	170	−2	< 0.001	< 0.001
RNA_PROCESSING	125	−2	< 0.001	< 0.001
DNA_DIRECTED_RNA_POLYMERASEII_HOLOENZYME	51	−2	< 0.001	< 0.001
SMALL_CONJUGATING_PROTEIN_LIGASE_ACTIVITY	40	−2	< 0.001	< 0.001
MITOSIS	68	−2	< 0.001	< 0.001
M_PHASE_OF_MITOTIC_CELL_CYCLE	71	−2	< 0.001	< 0.001
MRNA_PROCESSING_GO_0006397	47	−2	< 0.001	< 0.001
CHROMOSOMAL_PART	73	−2	< 0.001	< 0.001
CHROMOSOME_ORGANIZATION_AND_BIOGENESIS	97	−2	< 0.001	< 0.001
CHROMOSOME	94	−2	< 0.001	< 0.001
NUCLEAR_LUMEN	310	−2	< 0.001	< 0.001
CELL_CYCLE_PROCESS	154	−2	< 0.001	< 0.001

^a^ BAS *B. subtilis* DSM25841, CO Control

**Table 9:** Effect of the dietary supplementations of antibiotic on the transcriptomic profile of jejunum mucosa of weaned piglet 14 days after the *Escherichia coli* F4ac challenge

Name of the enriched gene set	Size, No. of genes	NES	*P*-value	FDR, *q*
Gene sets enriched in AB respect to CO^a^				
LYMPHOCYTE_ACTIVATION	50	2.22	< 0.001	< 0.001
LEUKOCYTE_ACTIVATION	56	2.2	< 0.001	< 0.001
IMMUNE_SYSTEM_PROCESS	260	2.17	< 0.001	< 0.001
IMMUNE_RESPONSE	182	2.3	< 0.001	< 0.001
CELLULAR_DEFENSE_RESPONSE	36	2.23	< 0.001	< 0.001
T_CELL_ACTIVATION	38	2.02	< 0.001	< 0.001
CELL_ACTIVATION	61	2.05	< 0.001	< 0.001
LYMPHOCYTE_DIFFERENTIATION	23	2.02	< 0.001	< 0.001
DEFENSE_RESPONSE	183	1.96	< 0.001	0.01
B_CELL_ACTIVATION	16	1.92	< 0.001	0.01
REGULATION_OF_IMMUNE_SYSTEM_PROCESS	53	1.88	< 0.001	0.02
POSITIVE_REGULATION_OF_IMMUNE_SYSTEM_PROCESS	42	1.87	< 0.001	0.02
HEMOPOIETIC_OR_LYMPHOID_ORGAN_DEVELOPMENT	67	1.86	< 0.001	0.02
LEUKOCYTE_DIFFERENTIATION	33	1.86	< 0.001	0.02
INTERLEUKIN_RECEPTOR_ACTIVITY	17	1.84	0.003	0.03
INTERLEUKIN_BINDING	22	1.82	0.003	0.03
T_CELL_DIFFERENTIATION	15	1.82	0.005	0.03
REGULATION_OF_LYMPHOCYTE_ACTIVATION	30	1.82	< 0.001	0.03
IMMUNE_SYSTEM_DEVELOPMENT	70	1.83	< 0.001	0.03
PROTEIN_KINASE_CASCADE	227	1.81	< 0.001	0.03
Gene sets enriched in CO respect to AB^a^				
AMINE_TRANSMEMBRANE_TRANSPORTER_ACTIVITY	32	−2.2	< 0.001	0.01
L_AMINO_ACID_TRANSMEMBRANE_TRANSPORTER_ACTIVITY	15	−2	< 0.001	0.05
CARBOXYLIC_ACID_TRANSMEMBRANE_TRANSPORTER_ACTIVITY	35	−2	< 0.001	0.05

^a^AB Antibiotic, CO Control

The comparison between BAA and CO groups showed 40 gene sets significantly enriched for the BAA group and 200 gene sets enriched for the CO group (FDR % < 25 and *P*-values of NES < 0.05). In the BAA group, gene sets related to cell energy metabolism including mitochondrial (MITOCHONDRIAL_MEMBRANE_PART, MITOCHONDRIAL_RESPIRATORY_CHAIN and MITOCHONDRIAL_INNER_MEMBRANE), and ribosomal (STRUCTURAL_CONSTITUENT_OF_RIBOSOME and RIBOSOME) activity were enriched; while the CO group showed enriched gene set related to cell proliferation, DNA and RNA activities (EXONUCLEASE_ACTIVITY, HELICASE_ACTIVITY, MEIOTIC_CELL_CYCLE, CELL_ CYCLE_PHASE, DNA_REPAIR, CELL_CYCLE_PROCESS) (Table 7).

The comparison between BAS and CO groups showed 131 gene sets significantly enriched for the BAA group and 303 gene sets enriched for the CO group (FDR % < 25 and *P*-values of NES < 0.05). In the BAS group, enriched gene sets involved cell signalling (RHODOPSIN_LIKE_RECEPTOR_ACTIVITY, CELL_CELL_SIGNALING, SUBSTRATE_SPECIFIC_CHANNEL_ACTIVITY, G_PROTEIN_COUPLED_RECEPTOR_ACTIVITY, G_PROTEIN_SIGNALING_COUPLED_TO_CYCLIC_NUCLEOTIDE_SECOND_MESSENGER) and cell transporters (MONOVALENT_INORGANIC_CATION_TRANSPORT, SODIUM_ION_TRANSPORT, CATION_CHANNEL_ACTIVITY, ION_CHANNEL_ACTIVITY) ranked the top; while the CO group showed enriched gene set related to cell proliferation, mitotic and meiotic functions (MICROTUBULE_ORGANIZING_CENTER, CENTROSOME, MITOSIS) (Table 8).

The comparison between AB and CO groups showed 141 gene sets significantly enriched for the AB group and 53 gene sets enriched for the CO group (FDR % < 25 and *P*-values of NES < 0.05). In the AB group, gene sets linked with immune response (LYMPHOCYTE_ACTIVATION, LEUKOCYTE_ACTIVATION, IMMUNE_SYSTEM_PROCESS, T and B CELLS ACTIVATION) ranked the top, while in the CO group several gene sets related to cell trans-membrane-transport (AMINE_TRANSMEMBRANE_TRANSPORTER_ACTIVITY, L_AMINO_ACID_TRANSMEMBRANE_TRANSPORTER_ACTIVITY and CARBOXYLIC_ACID_TRANSMEMBRANE_TRANSPORTER_ACTIVITY) ranked the top (Table 9).

**Fig. 1:**
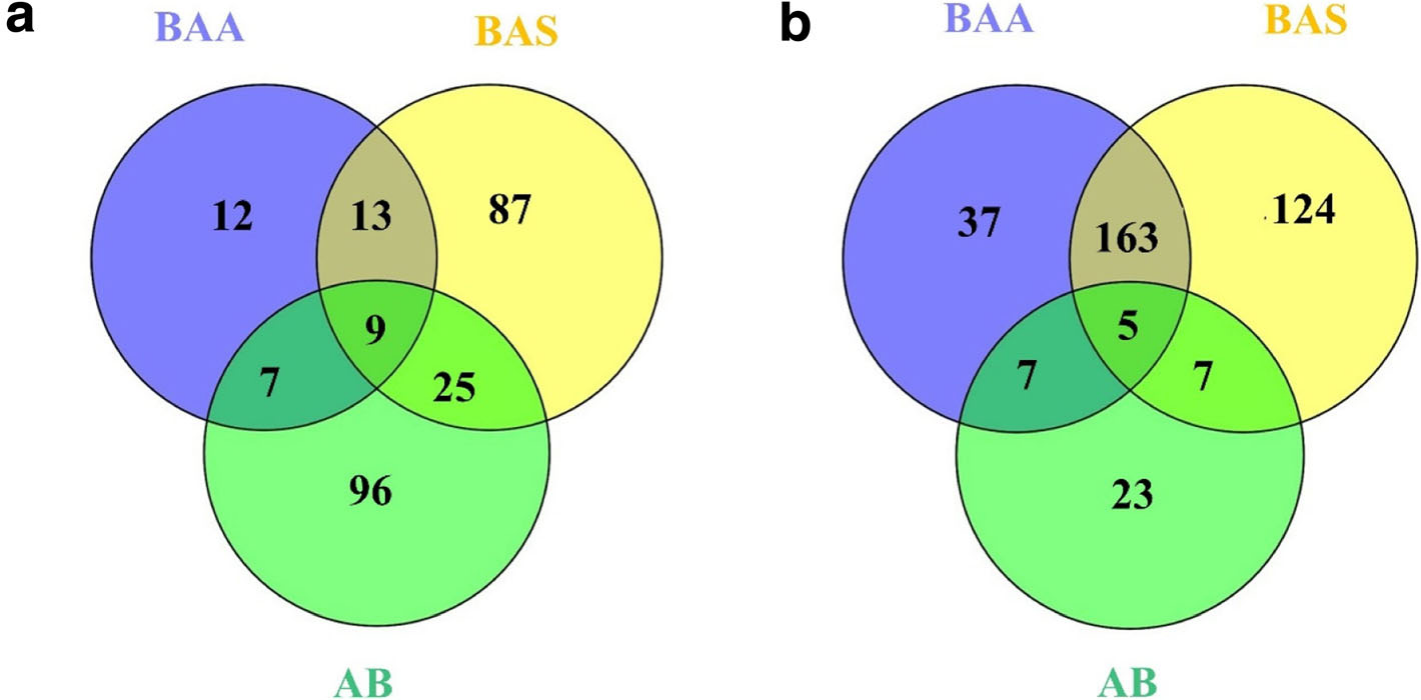
Numbers of commonly up-regulated (**a**) and down-regulated (**b**) gene sets (FDR < 0.25) among BAA, BAS and AB groups, compared with the CO group. 9 Gene sets of intersection AB|BAA|BAS were up-regulated: LYMPHOCYTE_ACTIVATION, LEUKOCYTE_ACTIVATION, T_CELL_ACTIVATION, LYMPHOCYTE_DIFFERENTIATION, B_CELL_ACTIVATION, T_CELL_DIFFERENTIATION, CELL_STRUCTURE_DISASSEMBLY_DURING_APOPTOSIS, DETECTION_OF_EXTERNAL_STIMULUS, DETECTION_OF_STIMULUS. 5 Gene sets of intersection AB|BAA|BAS: TRANSMEMBRANE_RECEPTOR_PROTEIN_KINASE_ACTIVITY, TRANSMEMBRANE_ RECEPTOR_PROTEIN_TYROSINE_KINASE_ACTIVITY, ENDOMEMBRANE_SYSTEM, CELL_MATRIX_ADHESION, CELL_SUBSTRATE_ADHESION

Overlaps of up and down-regulated gene sets between BAA, BAS and AB compared to CO are visualized in Venn Plot (Fig. 1). Nine gene sets were commonly up-regulated in BAA, BAS and AB groups compared to the CO group: LYMPHOCYTE_ACTIVATION, LEUKOCYTE_ACTIVATION, T_CELL_ACTIVATION, LYMPHOCYTE_DIFFERENTIATION, B_CELL_ACTIVATION, T_CELL_DIFFERENTIATION, CELL_STRUCTURE_DISASSEMBLY_DURING_APOPTOSIS, DETECTION_OF_EXTERNAL_STIMULUS, DETECTION_OF_STIMULUS. Five gene set were commonly down-regulated in BAA, BAS and AB groups compared to the CO group: TRANSMEMBRANE_RECEPTOR_PROTEIN_KINASE_ACTIVITY, TRANSMEMBRANE_ RECEPTOR_PROTEIN_TYROSINE_KINASE_ACTIVITY, ENDOMEMBRANE_SYSTEM, CELL_MATRIX_ADHESION, CELL_SUBSTRATE_ADHESION.

#### Morphometric, histochemical and immunohistochemistry

**Fig. 2:**
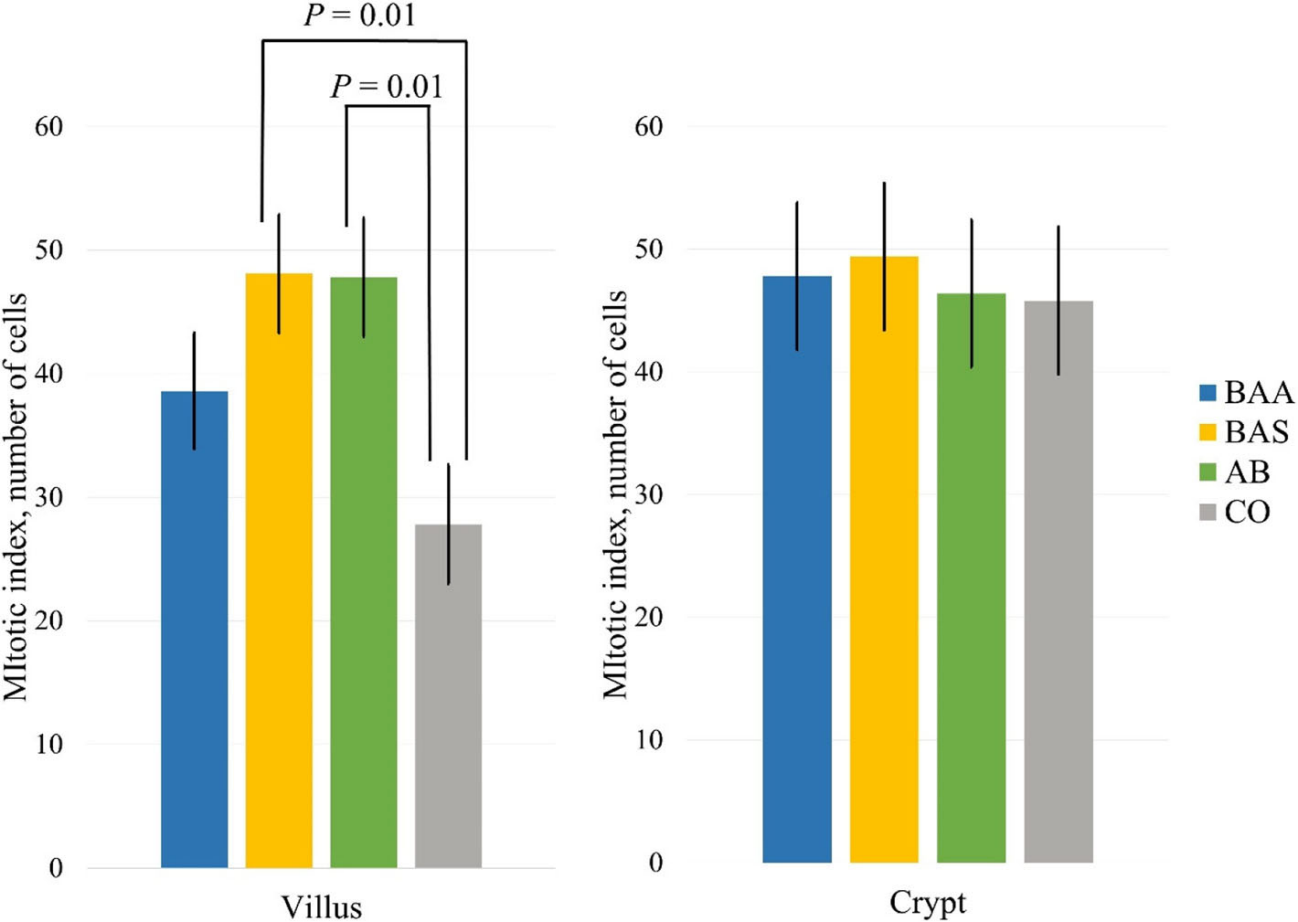
Effect of the dietary supplementations of *Bacillus amyloliquefaciens* DSM25840, *Bacillus subtilis* DSM25841 and antibiotic on jejunal villi and crypt mitotic index of weaned piglets 14 days after the ETEC F4ac challenge. BAS and AB had a higher mitotic index in the jejunal villi than CO (*P* = 0.01). BAA: *B. amyloliquefaciens* DSM25840; BAS*: B. subtilis* DSM25841; AB: Antibiotic; CO: Control

No significant differences were observed among the experimental groups for villus and crypt morphology, and for the number of PAS and ALB goblet cells at villus level (Additional file 4: Table S3; SS3). The number of PAS-ALB cells in the crypt was significantly lower in the AB and BAA groups than in the CO group (7.42 n./crypt in BAA; 7.42 n./crypt in AB; 8.90 n./crypt in CO; *P* = 0.05), while no difference was observed for the counts of PAS and ALB positive mucin cells (Table SS3). No differences were observed for the apoptotic index and for the number of IgA positive cells among the experimental groups. The mitotic index in villi of jejunum was lowest in the CO group than in the BAS and AB groups respectively (*P* = 0.01), while no difference among groups was observed for the mitotic index in the crypt (Fig. 2).

## Discussion

The present study explores the effect of *B. amyloliquefaciens* DSM25840 or *B. subtilis* DSM25841 supplementation in the feed of post-weaning pigs in order to prevent the detrimental effect of ETEC F4ac in subjects genetically susceptible and challenged with this pathogen. A group treated with colistin was used as a positive reference due to the sensitivity of *E. coli* strain used in this trial to this antibiotic [1]. Colistin confirms its capacity to contrast the ETEC F4 infection by reducing the number of piglets excluded from the trial for severe impairment, reducing the piglets’ fecal scores and by improving piglet growth performance compared to the CO group. Supplementation with *B. subtilis* was able to ameliorate the ETEC infection as shown by the reduced fecal scores compared to the CO group one week after the ETEC infection. Previous observation of Hu et al. [17] showed that different doses (2 × 10^9^ or 4 × 10^9^ or 20 × 10^9^ CFU/kg feed) of *B. subtilis* KN-42 reduced diarrhea index and fecal *E. coli* excretion in non-challenged post-weaning pigs, thus our study confirms that *B. subtilis* supplementation may reduce diarrhea due to ETEC in post-weaning pigs. On the other hand, *B. amyloliquefaciens* showed a scarce protective capacity against the ETEC F4ac infection, since no difference in fecal score consistency compared to the CO group was observed. From the authors knowledge, few data are published on the effect of *B. amyloliquefaciens* supplementation to counteract the ETEC infection in piglets *in vivo*. Our results could not confirm the observation of Ji et al. [11] where *B. amyloliquefaciens* SC06 increased the pro-inflammatory immune response (IL-6, IL-8, TNF-α, and IL-1α) in an *in vitro* system based on ETEC challenge. Moreover, in the same work, *B. amyloliquefaciens* SC06 decreased the diarrhea incidence by 79.2% in non-infected post-weaning pigs.

In the present study, blood parameters were primarily analysed in order to investigate differences among groups during the acute post-infection phase. A target metabolomics approach was applied to detect metabolic differences and biochemical mechanisms affecting the health status of pigs [35]. Dietary treatments particularly influenced the profile of plasma amino acids, whose concentrations were higher in the CO group. This general increase of plasma amino acid concentrations in the CO group, and especially the higher level of lysine, can be explained by a transient increase of catabolic insult leading to muscle breakdown and nitrogen loss as reported by Freund et al. [36] in septiceps patients. Furthermore, according to Rochell et al. [37], we can also hypothesize that plasma amino acids increased as their demand for skeletal muscle synthesis was reduced in CO group compared to other experimental groups. Indeed, Rochell et al. [37] showed that even if the digestibility of birds infected with *Eimeria* was decreased compared to the non-infected animals, the plasma amino acids were not decreased homogeneously, suggesting that the level of amino acids in plasma was not directly referred to the absorption and the digestibility while it can be influenced by the skeletal muscle synthesis.

Furthermore, particular attention should be given to the higher level of plasma arginine in the CO group than in the AB and the *B. amyloliquefaciens* groups. Arginine is a semi-essential amino acid in pigs and it is an important initiator of immune response as well as a precursor of several metabolic pathways [38]. Particularly it acts as a substrate for enzymatic generation of the antimicrobial molecule nitric oxide (NO), a radical that can inhibit bacterial respiration, DNA replication and modify specific metabolic pathways including the tricarboxylic acid cycle [38]. It can be assumed that the higher level of serum arginine in CO group could be associated with a lower hepatic degradation through the urea cycle, in order to limit the loss of body nitrogen (N) and the overburden use of essential amino acids [39]. Though, Thomsen et al. [40] showed that in acute phase response the urea cycle enzymes at mRNA levels decreased immediately but gradually returned stable resulting in no change of cycle enzyme proteins, in the present study, it is possible that this preventive control of acute phase condition was not effective in CO group. Furthermore, arginine turns over rapidly in mammals as inside cells multiple pathways are involved in arginine degradation to produce NO, ornithine, urea, polyamines, proline, glutamate, creatine [41]. In the present study, both ornithine and proline were numerically higher in CO group with respect to the *Bacillus* sp. groups, suggesting that, in the CO group part of arginine was already degraded involving ornithine and proline pathways. Interestingly, a reduced level of glycine and serine was observed in the BAS group which may be associated with the utilization of these amino acids by the *B. subtilis* strains during sporulation as observed by Mitani and Kadota [42].

In addition to plasma metabolites, the animal health condition was evaluated by analysis of serum acute phase proteins 24 h after the ETEC infection. CRP, SAA and Hp are generally considered inflammatory markers; their production by the liver is stimulated by cytokines released from activated phagocytes. In the present study, CRP and SAA were influenced neither by *Bacillus* administration nor by antibiotic supplementation, however, a previous study suggested that 24 h post-F4ac ETEC infection could be not sufficient to rise CRP level [5]. On the other hand, Hp was reduced in the AB group compared to the CO group, confirming a higher stimulation of inflammatory response in control animals.

In the present study, piglets were then followed until 14 days’ post-infection when they recovered from the infection (as confirmed by their reduced fecal score).

Studies suggested that dietary *Bacillus* spp. supplementation can influence gut microbial profile by promoting the development of beneficial bacteria and by having a competitive action against pathogens as *Bacillus* spp. can produce a wide variety of antimicrobial compounds [18, 43]. In the present study, no significant differences in alpha and beta diversity indices were observed among groups both in jejunum and in cecum. The presented results are not in accordance with other previous findings in which bacterial communities have been modified by *Bacillus* spp. supplementation [10, 17, 19]; however, divergent results could be due to different experimental conditions among studies and different microbiota profiles prior to the experiment. Nevertheless, a trend of lower Enterobacteriaceae abundancy in the caecum content was observed both in *Bacillus* and in AB treatments compared to the CO group. Enterobacteriaceae family includes both non-pathogenic (commensal bacteria) and pathogens such as ETEC [44]. Among the wide amount of bacteria family present in the gut, Enterobacteriaceae family has been proposed as a non-invasive biomarker of intestinal health [45], since an increase of Enterobacteriaceae has been associated whit intestinal dysbiosis in mice, poultry and weaning pigs [46–48]. It cannot directly be asserted that the probiotics reduced ETEC prevalence, but a reduction in the Enterobacteriaceae abundance could contribute to decreasing the risk of pathogens development, as previously observed for other probiotics such as *Lactobacillus (L.) plantarum* 4.1 and *L. reuteri* 3S7 [49] and *L. salivarius* DPC6005, *Pediococcus pentosaceus* DPC6006 and *L. pentosus* DPC6004 [50]. Furthermore, presence and/or differences in abundance of few bacteria in the gut can be sufficient to modify the gene expression regulation in the intestine [51]. In the present study, despite a limited difference in the microbiota profile among groups was observed, it was enough to influence the jejunum transcriptomic profile. The presented results showed that both *Bacillus* strains and AB supplementations up-regulated several gene sets related to immune response, including gene sets involved in the stimulus’ detection and in adaptive immune response capability (B and T cells lymphocyte activation). Antibiotic supplementation showed the highest stimulation of gene set for lymphocyte activation and differentiation followed by *B. subtilis* supplementation, while a less intense up-regulation of these gene sets was observed in the *B. amyloliquefaciens* supplementation group. These results indicate that pigs supplied with *Bacillus* spp. and antibiotic recovered faster than pigs in the CO group, which is supported by the finding that the cell mitotic index was improved in jejunum villi in both AB and *B. subtilis* groups*.*

In addition, compared to antibiotic administration, the supplemented *B. subtilis* exerted a stimulation on the immune response supported by the observed higher blood IgA level [52], which is consistent with the results of Guo et al. [53].

On the contrary, the CO group showed a higher stimulation of gene sets related to nuclear structure, RNA processing and of other gene sets related to cell mitosis (microtubule, centrosome) which indicates an improvement of intestinal cells turnover in control animals compared to the other groups and it could be due to a previous higher injury in CO group [54]. These observations at mRNA level were not traduced into a concrete animal recovery as indicated by the results for blood and immunohistochemistry parameters. Thus, it can be hypothesized that animals in the CO group had a slower recovery phase compared to pigs in the AB and BAS groups. The slower and reduced recovery capacity of CO group with respect to both AB and BAS groups are also supported by the higher number of animals excluded from the trial for their severe health impartments.

Furthermore, gene sets related to anion, cation, sodium and potassium channel activation, which are all associated with the intestinal absorption and secretion activities [55] were stimulated by *B. subtilis*. The intestinal stimulation of gene sets related to absorption and secretion indicates improved nutrients utilization lending a better feed transformation into body mass and improved animals performance [15]. Thus, the results of the present study support the hypothesis that the probiotic effect of *Bacillus* strains tested in the present trial can be ascribed to an improvement of feed digestibility by their enzymatic activity [56, 57]. This has also been confirmed by *in vitro* data showing secretion of NSP-enzymes and proteases by DSM25840 and DSM25841 [8] and by *in vivo* studies showing improved protein digestibility when these two strains were fed to grower-finisher pigs [58]. Despite an improvement of growth performance in probiotic groups was therefore expected in the present study, this result may be lack because only the most performing pigs were kept in the CO group. Indeed, a higher number of animals were excluded for severe health impairment in the CO group compared to the other groups. Thus it cannot be excluded a potential favouring influence of *Bacillus* spp. on pigs’ performance.

## Conclusion

The presented data provide an exhaustive picture on the effectiveness of the tested strain *B. subtilis* DSM25841 to mitigate the detrimental effect of ETEC F4ac infection in weaned pigs susceptible to this pathogen. *B. subtilis* promoted the piglet gut health by reducing the Enterobacteriaceae level, favouring the up-regulation of genes related to immunity, contributing in a higher immune competence and in an improvement of the amino acids metabolism and utilization. These are the rationales for favouring the animal post-infection recovery, confirmed by the lower number of pigs with severe diarrhea observed during the study in the treated groups.

## Additional files

Additional file 1: **Table S1.** List of the quantified metabolites using the MS/MS targeted analysis. (CSV 18 kb)

Additional file 2: **Table S2.** List of number of Observed OTUs, Shannon index and number of reads in jujunum and cecum samples. (DOCX 34 kb)

Additional file 3: **Figure S1.** Non-Metric Multidimensional Scaling (NMDS) plot on Bray-Curtis distance matrix in jejunum and cecum of ETEC F4-infected pigs fed supplementations of *Bacillus amyloliquefaciens* DSM25840, *Bacillus subtilis* DSM25841 and antibiotic at d 21. (DOCX 521 kb)

Additional file 4: **Table S3.** Effect of the dietary supplementations of *Bacillus amyloliquefaciens* or *Bacillus subtilis*, on the jejunum histology and morphology of weaned pigs 14 days post-infection with ETEC F4. (DOCX 39 kb)

## Data Availability

All data generated or analysed during this study are included in this published article [and its supplementary information files]. The datasets on microbiota data supporting the conclusions of this article are available in the PiGutNet area of the European Nucleotide Database (ENA) repository with number ERP114115.

## Abbreviations

ADFI: Average daily feed intake
ADWG: Average daily weight gain
BW: Body weight
CFU: Colony-forming unit
*E. coli*: *Escherichia coli*
ETEC: Enterotoxigenic *Escherichia coli*
F:G: Feed to gain ratio
Igs: Immunoglobulins
LAB: Lactic acid bacteria
PWD: Post-weaning diarrhea
PWDS: Post-weaning diarrhea syndrome
