# Supplementary material for: Bacillus sp. probiotic supplementation diminish the Escherichia coli F4ac infection in susceptible weaned pigs by influencing the intestinal immune response, intestinal microbiota and blood metabolomics
Source: J Anim Sci Biotechnol. 2019 Sep 12;10:74. doi: 10.1186/s40104-019-0380-3 (PMC6740008; doi:10.1186/s40104-019-0380-3)
Supplement: Supplementary file 2 — Table S2. List of number of Observed OTUs, Shannon index and number of reads in jujunum and cecum samples. (DOCX 34 kb) [file 40104_2019_380_MOESM2_ESM.docx]

Supplementary Table 2. List of number of Observed OTUs, Shannon index and number of reads in jujunum and cecum samples

| ID | Treatment^a^ | Observed OTUs | Shannon | No. of reads |
| --- | --- | --- | --- | --- |
| Jejunum |  |  |  |  |
| 63469 | BAA | 180 | 2.665 | 11660 |
| 59863F52349 | BAA | 199 | 1.961 | 34831 |
| 63464 | BAA | 128 | 1.902 | 28079 |
| 63471 | BAA | 110 | 1.989 | 11059 |
| 59879F52384 | BAS | 269 | 3.154 | 22989 |
| 63486 | BAS | 197 | 2.584 | 13026 |
| 63459 | BAS | 282 | 4.083 | 49244 |
| 63477 | BAS | 160 | 3.410 | 5610 |
| 59883F52392 | BAS | 163 | 1.744 | 21488 |
| 63488 | BAS | 135 | 2.446 | 8964 |
| 63484 | BAS | 188 | 2.146 | 50723 |
| 59869F52360 | BAS | 100 | 1.558 | 8265 |
| 63461 | BAS | 168 | 3.188 | 10043 |
| 63465 | BAS | 219 | 2.682 | 19350 |
| 59882F52391 | AB | 232 | 2.810 | 22375 |
| 59868F52358 | AB | 112 | 1.545 | 8749 |
| 63463 | AB | 140 | 2.994 | 5472 |
| 63475 | AB | 218 | 2.674 | 73065 |
| 59876F52379 | AB | 169 | 2.865 | 8148 |
| 63470 | AB | 129 | 2.513 | 5448 |
| 59884F52393 | CO | 170 | 1.739 | 23500 |
| 63489 | CO | 200 | 2.497 | 23766 |
| 59875F52378 | CO | 237 | 3.272 | 24338 |
| 63479 | CO | 161 | 2.596 | 12508 |
| Cecum |  |  |  |  |
| 59816F52320 | BAA | 454 | 3.496 | 11627 |
| 59829F52333 | BAA | 740 | 4.662 | 27637 |
| 59835F52339 | BAA | 548 | 3.772 | 31953 |
| 59790F52294 | BAA | 653 | 4.102 | 27129 |
| 63398F52304 | BAA | 759 | 3.997 | 36032 |
| 59811F52315 | BAA | 561 | 4.114 | 26591 |
| 59825F52329 | BAS | 445 | 3.530 | 27302 |
| 59789F52293 | BAS | 708 | 4.316 | 25264 |
| 59809F52313 | BAS | 739 | 4.382 | 20993 |
| 59836F52340 | BAS | 699 | 4.650 | 21829 |
| 59833F52337 | BAS | 424 | 3.125 | 27137 |
| 59801F52305 | BAS | 722 | 4.539 | 43074 |
| 59792F52296 | BAS | 712 | 4.362 | 29372 |
| 59802F52306 | BAS | 581 | 4.240 | 30266 |
| 59817F52321 | BAS | 743 | 4.666 | 27157 |
| 59807F52311 | AB | 591 | 4.171 | 30556 |
| 59832F52336 | AB | 545 | 4.672 | 9787 |
| 59799F52303 | AB | 483 | 3.818 | 16783 |
| 59826F52330 | AB | 680 | 4.764 | 49827 |
| 59828F52332 | AB | 683 | 4.607 | 39629 |
| 59791F52295 | AB | 629 | 3.976 | 26744 |
| 59818F52322 | AB | 620 | 3.802 | 32024 |
| 59820F52324 | AB | 596 | 4.551 | 15701 |
| 63400F52314 | AB | 459 | 2.990 | 10497 |
| 59798F52302 | CO | 644 | 4.396 | 19170 |
| 59834F52338 | CO | 896 | 5.035 | 36712 |
| 59819F52323 | CO | 465 | 4.057 | 15381 |
| 59793F52297 | CO | 722 | 4.488 | 26139 |
| 59808F52312 | CO | 551 | 4.637 | 10761 |
| 63401F52331 | CO | 633 | 4.126 | 25552 |

^a^ BAA: *B. amyloliquefaciens*; BAS*: B. subtilis*; AB: Antibiotic; CO: Control.
