# Supplementary material for: Bacillus sp. probiotic supplementation diminish the Escherichia coli F4ac infection in susceptible weaned pigs by influencing the intestinal immune response, intestinal microbiota and blood metabolomics
Source: J Anim Sci Biotechnol. 2019 Sep 12;10:74. doi: 10.1186/s40104-019-0380-3 (PMC6740008; doi:10.1186/s40104-019-0380-3)
Supplement: Supplementary file 3 — Figure S1. Non-Metric Multidimensional Scaling (NMDS) plot on Bray-Curtis distance matrix in jejunum and cecum of ETEC F4-infected pigs fed supplementations of Bacillus amyloliquefaciens DSM25840, Bacillus subtilis DSM25841 and antibiotic at d 21. (DOCX 521 kb) [file 40104_2019_380_MOESM3_ESM.docx]

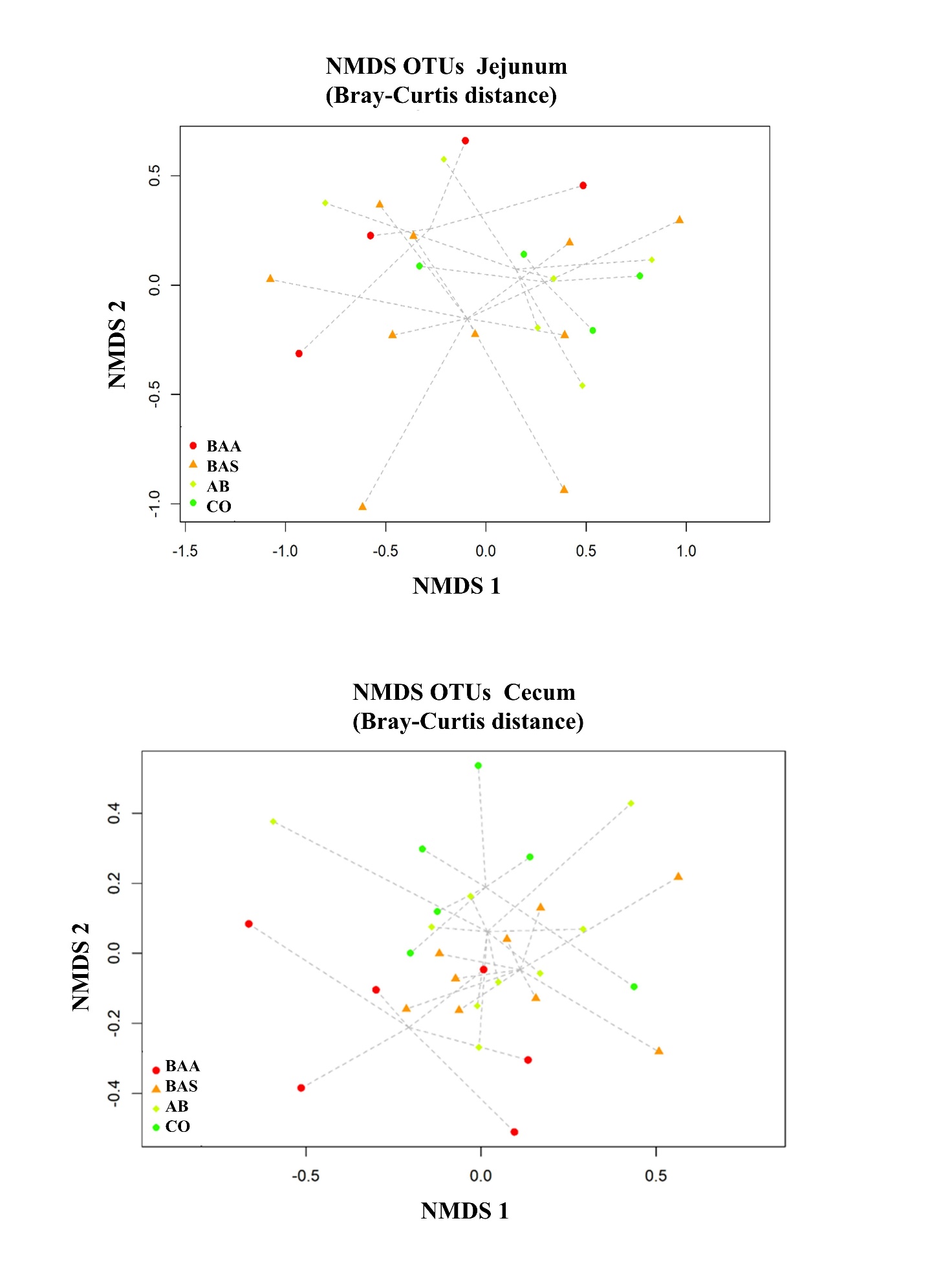


Supplementary Fig. 1. Non-Metric Multidimensional Scaling (NMDS) plot on Bray-Curtis distance matrix in jejunum and cecum of ETEC F4-infected pigs fed supplementations of *Bacillus amyloliquefaciens* DSM25840, *Bacillus subtilis* DSM25841 and antibiotic at d 21.

BAA= *B. amyloliquefaciens*; BAS= *B. subtilis*; AB= Antibiotic; CO= Control.
