# Supplementary material for: Bacillus sp. probiotic supplementation diminish the Escherichia coli F4ac infection in susceptible weaned pigs by influencing the intestinal immune response, intestinal microbiota and blood metabolomics
Source: J Anim Sci Biotechnol. 2019 Sep 12;10:74. doi: 10.1186/s40104-019-0380-3 (PMC6740008; doi:10.1186/s40104-019-0380-3)
Supplement: Supplementary file 4 — Table S3. Effect of the dietary supplementations of Bacillus amyloliquefaciens or Bacillus subtilis, on the jejunum histology and morphology of weaned pigs 14 days post-infection with ETEC F4. (DOCX 39 kb) [file 40104_2019_380_MOESM4_ESM.docx]

Supplementary Table 3. Effect of the dietary supplementations of *Bacillus amyloliquefaciens* and *Bacillus subtilis*, on the jejunum histology and morphology of weaned pigs 14 days post-infection with ETEC F4.

| Items | Treatments* | | | | SEM | *P*- value | | |
| --- | --- | --- | --- | --- | --- | --- | --- | --- |
|  | BAA | BAS | AB | CO |  | BAA vs. CO | BAS vs. CO | AB vs. CO |
| Villus, µm | | | | | | | | |
| Height | 354.2 | 407.8 | 378.5 | 372.8 | 17.6 | 0.48 | 0.19 | 0.83 |
| Width | 120 | 146.3 | 144.2 | 125.8 | 8.2 | 0.63 | 0.11 | 0.14 |
| Crypt, µm | | | | | | | | |
| Width | 47.7 | 53.4 | 50.5 | 50.3 | 2.2 | 0.43 | 0.35 | 0.93 |
| Depth | 287.4 | 268.3 | 254.2 | 296.2 | 19.4 | 0.76 | 0.34 | 0.15 |
| Mucosal surface area, M index† | 6.6 | 6.49 | 6.38 | 6.68 | 0.45 | 0.91 | 0.8 | 0.65 |
| Mitotic index, No. of cells | | | | | | | | |
| Villus | 38.6 | 48.1 | 47.8 | 27.8 | 4.7 | 0.14 | 0.01 | 0.01 |
| Crypt | 47.8 | 49.4 | 46.4 | 45.8 | 6 | 0.82 | 0.68 | 0.95 |
| Apoptotic index, No. of cells | | | | | | | | |
| Villus | 0.6 | 0.6 | 0.7 | 0.5 | 0.2 | 0.68 | 0.61 | 0.49 |
| IgA positive cells, No. per 10,000 µm^2^ | | | | | | | | |
| Villus | 0.66 | 0.73 | 1.53 | 1.75 | 0.7075 | 0.31 | 0.34 | 0.83 |
| Crypt | 18.71 | 22.7 | 19.24 | 20.06 | 3.1025 | 0.77 | 0.57 | 0.86 |
| Goblet cells, No. /villus | | | | | | | | |
| PAS positive | 3.09 | 3.78 | 3.09 | 3.23 | 0.795 | 0.91 | 0.64 | 0.91 |
| Alcian positive | 3.3 | 4.51 | 3.22 | 2.86 | 0.9725 | 0.76 | 0.26 | 0.8 |
| PAS-ALC positive | 1.18 | 1.69 | 1.25 | 1.37 | 0.43 | 0.77 | 0.62 | 0.85 |
| Goblet cells, No. /crypt | | | | | | | | |
| PAS positive | 14.33 | 15.68 | 11.38 | 15.13 | 2.35 | 0.82 | 0.88 | 0.28 |
| Alcian positive | 7.48 | 7.52 | 10.12 | 7.85 | 1.36 | 0.86 | 0.87 | 0.26 |
| PAS-ALC positive | 7.42 | 8.66 | 7.42 | 8.9 | 0.47 | 0.05 | 0.73 | 0.05 |

*BAA: *B*. *amyloliquefaciens*; BAS: *B*. *subtilis*; AB: Antibiotic; CO: Control

^†^Calculated as described by Kisielinski et al. [1].

**References**

[1] Kisielinski K, Willis S, Prescher A, Klosterhalfen B, Schumpelick V. A simple new method to calculate small intestine absorptive surface in the rat. Clin. Exp. Med. 2002; 2: 131-135.
